# Mangostanin, a Xanthone Derived from *Garcinia mangostana* Fruit, Exerts Protective and Reparative Effects on Oxidative Damage in Human Keratinocytes

**DOI:** 10.3390/ph15010084

**Published:** 2022-01-11

**Authors:** Mario Abate, Cristina Pagano, Milena Masullo, Marianna Citro, Simona Pisanti, Sonia Piacente, Maurizio Bifulco

**Affiliations:** 1 Department of Medicine and Surgery, University of Salerno, 84081 Baronissi, Italy; mabate@unisa.it (M.A.); mcitro@unisa.it (M.C.); 2 Department of Molecular Medicine and Medical Biotechnology, University of Naples “Federico II”, 80131 Naples, Italy; pagano.cris@gmail.com; 3 Department of Pharmacy, University of Salerno, 84084 Fisciano, Italy; mmasullo@unisa.it (M.M.); piacente@unisa.it (S.P.)

**Keywords:** mangostanin, *Garcinia mangostana*, oxidative stress, apoptosis, cosmeceuticals

## Abstract

The fruit of *Garcinia mangostana* (mangosteen) is known in ancient traditional Asian medicine for its antioxidant, anti-inflammatory, immunomodulatory and anticancer activities. These effects are mainly due to the action of polyphenols known as xanthones, which are contained in the pericarp of the fruit. In recent years, there has been a growing interest from pharmaceutical companies in formulating new topicals based on mangosteen full extracts to prevent skin aging. However, the molecules responsible for these effects and the mechanisms involved have not been investigated so far. Here, the arils and shells of *Garcinia mangostana* were extracted with chloroform and methanol, and the extracts were further purified to yield 12 xanthone derivatives. Their effects were evaluated using in vitro cultures of human epidermal keratinocytes. After confirming the absence of cytotoxicity, we evaluated the antioxidant potential of these compounds, identifying mangostanin as capable of both protecting and restoring oxidative damage induced by H_2_O_2_. We showed how mangostanin, by reducing the generation of intracellular reactive oxygen species (ROS), prevents the activation of AKT (protein kinase B), ERK (extracellular signal-regulated kinase), p53, and other cellular pathways underlying cell damage and apoptosis activation. In conclusion, our study is the first to demonstrate that mangostanin is effective in protecting the skin from the action of free radicals, thus preventing skin aging, confirming a potential toward its development in the nutraceutical and cosmeceutical fields.

## 1. Introduction

*Garcinia mangostana* L. (mangosteen) is an evergreen tropical tree native to Southeast Asia belonging to the Clusiaceae or Guttiferae family [1]. It is renowned as the “queen of the fruits” due to the particularly delicious taste of the fruits as well as for its use in traditional medicine [2]. In fact, in Southeast Asia, the seeds and peels have been used not only as infusions and decoctions for the treatment of gastrointestinal and urinary tract infections but also as a laxative, anti-fever agent, and an insomnia treatment [2,3].

Various components have been found to be responsible for the pharmacological properties of mangosteen, such as saccharides, flavonoids, phenolic acids, and, most importantly, xanthones.

Plant-derived polysaccharides have been reported to possess a wide range of biological activities, such as anti-inflammatory and immunoregulatory activity. Furthermore, they can be easily used in the biomedical field as they are non-toxic and free from apparent side effects. Recently, a water-soluble arabinofuranan was purified from the rinds of *Garcinia mangostana* and was reported to have a strong stimulating effect on the immune system, particularly through the activation of macrophages [4].

Similarly, flavonoids are compounds often synthesized by plants, including mangosteen, as a defense against pathogens, such as *Xanthomonas campestris* and *Fusarium* species, nematodes, and phytophagous insects. Since their discovery, they have been described as having a vast number of biological benefits, such as anti-inflammatory, antiviral, antibacterial, antioxidant, anticancer, and neuroprotective properties. Baicalein, fisetin, quercetagetin, silymarin, curcumin, and nobiletin are just some examples of the numerous flavonoids that can be found in mangosteen; interestingly, various studies have reported that these flavonoids can inhibit the infection caused by chikungunya virus, a pathogen that can cause a fever characterized by severe myalgia [5,6].

Polyphenols, phytonutrients that have been shown to prevent cell degeneration and the proliferation of tumor cells, represent another important class of compounds that can be found in mangosteen. Several studies show how the implementation of diets rich in polyphenols of plant origin (such as mangosteen) can help avoid the development of tumors, especially in the stomach and surrounding areas. The group of flavonoids also includes catechins, moderate antioxidants that can help protect the cardiovascular system [7].

Despite the varied chemical composition of the mangosteen, many scientists are directing their research toward the isolation of the phytochemical compounds most represented in the mangosteen fruit, the xanthones, which may be responsible for its prodigious qualities. For the 68 xanthones that have been cataloged so far, multiple in vitro and in vivo experiments have been conducted with the aim of better understanding and identifying their multiple biological effects (antioxidant [8], antibacterial [9], anti-inflammatory [10,11], neuroprotective [12], hypoglycemic [13,14], anti-obesity [15], cytotoxic, anti-proliferative, and anti-cancer [16,17,18,19]). At the same time, several in vivo studies on mangosteen fruit extracts and its purified compounds, demonstrated the total absence of acute or chronic toxicity [20,21], confirming what has already been observed by the consumption of mangosteen for over 100 years.

For this reason, the use of bio-active extracts of mangosteen fruit is having great success in the nutraceutical and cosmetic fields. Recent studies show how mangosteen fruit extracts can be used as photoprotective agents from UVB damage [22], to improve skin elasticity, and to control inflammatory skin diseases [11,23]. However, although the extracts of mangosteen fruit are used for the preparation of cosmetics, the biological targets and the mechanism of action from which its dermatological benefits derive, are not yet clear.

Therefore, in this work we provided the first pharmacological evidence of how mangostanin, a xanthone present in the mangosteen extract, is able both to protect the skin from the deleterious action of free radicals preventing skin aging and to remedy to damage with a repairing effect on the skin after oxidative insult.

## 2. Results

### 2.1. Extraction, Characterization, and Purification of Twelve Compounds from GARCINIA Mangostana Fruit

**Figure 1:**
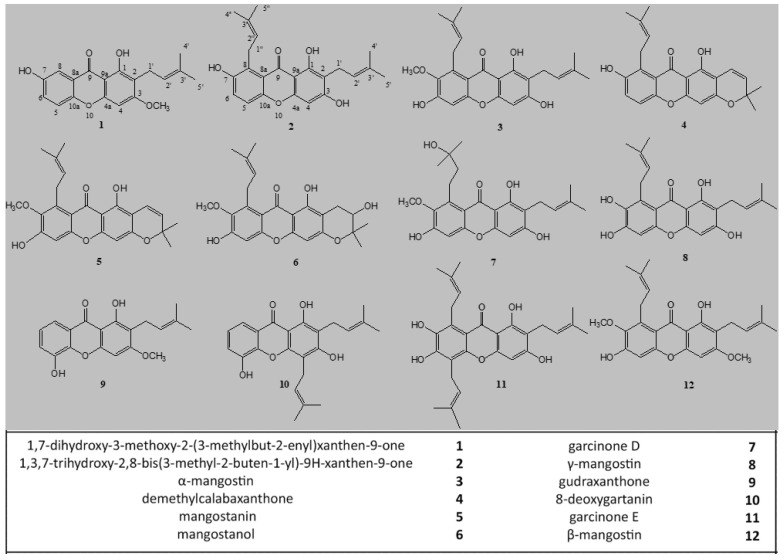
Chemical structures of xanthone derivatives (**1**–**12**) isolated from *Garcinia mangostana*.

**Figure 2:**
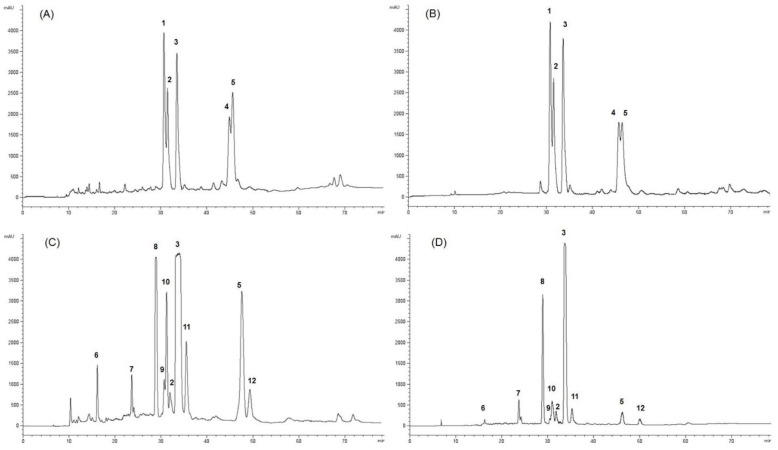
HPLC-UV chromatograms of *G. mangostana* extracts. (**A**) chloroform extract of arils, (**B**) methanol extract of arils, (**C**) chloroform extract of shells, and (**D**) methanol extract of shells.

The CHCl_3_ and MeOH extracts of *G. mangostana* arils (1 Ext CHCL3 and 1 Ext MeOH) and shells (2 Ext CHCL3 and 2 Ext MeOH) were further purified by HPLC-UV to afford compounds **1**–**12** (Figure 1). The chloroform and methanol extracts of mangosteen arils submitted to purification by HPLC-UV showed a similar profile characterized by the occurrence of five main compounds, identified as 1,7-dihydroxy-3-methoxy-2-(3-methylbut-2-enyl)xanthen-9-one (**1**), 1,3,7-trihydroxy-2,8-bis(3-methyl-2-buten-1-yl)-9H-xanthen-9-one (**2**), α-mangostin (**3**), demethylcalabaxanthone (**4**), and mangostanin (**5**), also known as 6-hydroxycalabaxanthone or 9-hydroxycalabaxanthone [2,24,25]. The chloroform extract of mangosteen shells submitted to purification by HPLC-UV allowed the isolation of mangostanol (**6**), garcinone D (**7**), γ-mangostin (**8**), gudraxanthone (**9**), 8-deoxygartanin (**10**), garcinone E (**11**), β-mangostin (**12**), along with compounds **2**, **3** and **5**. In the methanol extract of mangosteen shells, compounds **3**, **5**, and **8** represented the main compounds, while all the other compounds occurring in the chloroform extract of shells were in smaller quantities (Figure 2). Compounds **1**–**12** were recognized by NMR analysis to be xanthone derivatives [26,27]. Their structures were established by 1D-(^1^H and ^13^C) and 2D-NMR (DQF-COSY, HSQC, and HMBC) experiments. The ^13^C NMR values of compounds **1**–**12** are reported in the Table 1.

### 2.2. Evaluation of the Biological Effect of Garcinia mangostana Extracts and Isolated Compounds in Human Keratinocytes

**Figure 3:**
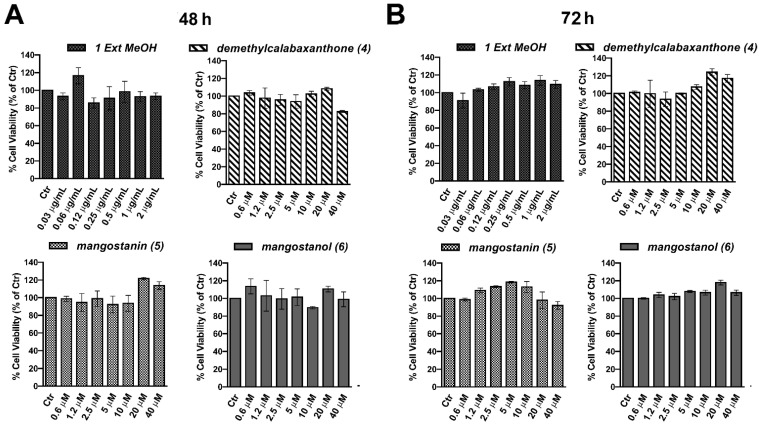
Evaluation of the methanol extract of *G. mangostana* arils and compounds 4–6 effects in HaCaT cells. Human keratinocytes were grown for 48 h (**A**) or 72 h (**B**) with individual compounds (0–40 μM) or extract (0–2 μg/mL) before MTT assay. Independent experiments were performed in triplicate, and the results are expressed as means ± SD and reported as % vs. the untreated cell (Ctr) (ANOVA).

First, we evaluated the effect of the four extracts and twelve compounds on human keratinocytes (HaCaT), a frequently employed skin model cell system. At first, we assessed the potential toxicity of both mangosteen extracts (0–2 μg/mL) and isolated compounds (0–40 μM). To this end, cells were grown with the substances for 48 h before evaluating cell viability through MTT assay (Supplementary Figure S1). In Figure 3A, we reported only the bar graphs relative to selected mangosteen methanol extract and the compounds demethylcalabaxanthone (**4**), mangostanin (**5**), and mangostanol (**6**). None of these showed an inhibitory effect on cell viability. Interestingly, we confirmed that even at 72 h, these compounds did not show any interference with cell viability, as shown in Figure 3B.

### 2.3. Mangostanin Reverts and Prevents Cytotoxicity Induced by H_2_O_2_ in HaCaT Cells

**Figure 4:**
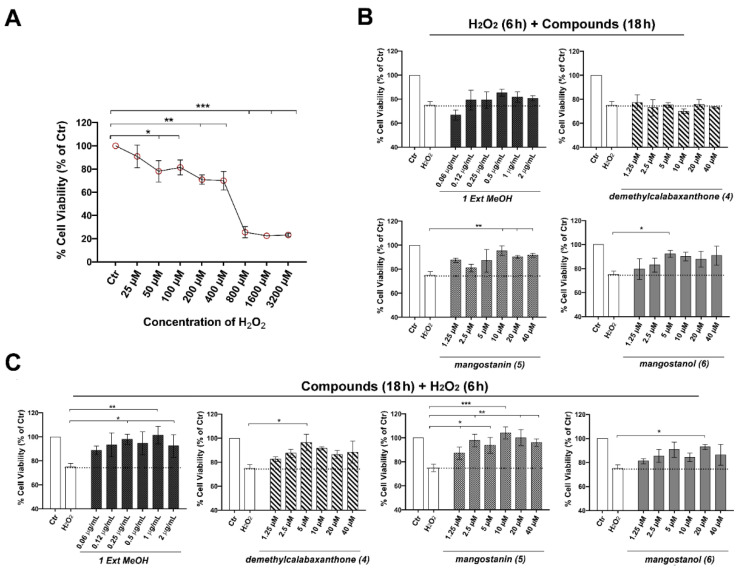
Mangostanin rescued and prevented H_2_O_2_-induced cytotoxicity in HaCaT cells. (**A**) Cells exposed to H_2_O_2_ (0–3200 μM) for 6 h before MTT assay. Results of three independent experiments performed in triplicate are expressed as means ± SD and reported as % vs. the untreated control (ANOVA, * *p* <0.05, ** *p* < 0.01, *** *p* < 0.001 vs. control). (**B**) Cells were grown in the presence of H_2_O_2_ for 6 h, before treatment with methanol extract of *G. mangostana* arils (0–2 μg/mL) or compounds demethylcalabaxanthone (**4**), mangostanin (**5**), mangostanol (**6**) at the indicated concentrations (0–40 μM) for 18 h. Results of independent experiments performed in triplicate are expressed as means ± SD and reported as % vs. the untreated control (ANOVA, * *p* < 0.05, ** *p* < 0.01 vs. control). (**C**) Keratinocytes were cultured in the presence of methanol extract of *G. mangostana* arils (0–2 μg/mL) or compounds 4–6 (0–40 μM) for 18 h, before treatment with H_2_O_2_ for 6 h. Results of independent experiments performed in triplicate are expressed as means ± SD and reported as % vs. the untreated control (ANOVA, * *p* <0.05, ** *p* < 0.01, *** *p* < 0.001 vs. control).

The next step was to evaluate both the potential reparative and preventive effect of the best mangosteen extract and selected compounds on the cell viability of HaCaT cells exposed to H_2_O_2_ (0–3200 μM) for 6 h as an inducer of cell damage. H_2_O_2_ induced cytotoxicity dose-dependently, starting at 50 μM, where the inhibition of cell viability was statistically significant, and reaching a reduction of 29.1% at 200 μM. Previous studies reported that lower concentrations of H_2_O_2_ (≤250 μM) induced apoptotic cell death of more cells, whereas higher H_2_O_2_ concentrations (1000 μM) resulted in an increase in overall cell death but a reduction in apoptosis [28,29]. Therefore, we decided to use 200 μM of H_2_O_2_ in the subsequent experiments. We observed that the pretreatment of keratinocytes with mangostanin in a concentration-dependent manner from 10 μM, and with mangostanol (5 μM), rescued the cell viability after H_2_O_2_-induced cytotoxicity. In detail, as shown in Figure 4B, survival rate, which dropped after exposure to H_2_O_2_ (200 μM for 6 h), increased to 95.45 ± 4.02%, 90.45 ± 1.14% and 91.81 ± 1.70% with mangostanin at 10, 20, and 40 μM, respectively. Furthermore, we evaluated the preventive effect of the same extract and compounds after 18-h pretreatment of HaCaT cells before H_2_O_2_-induced cell damage. We observed that the pretreatment with mangostanin from 2.5 to 40 μM was protective against H_2_O_2_-induced cytotoxicity. As shown in Figure 4C, cell viability declined after exposure to 200 μM of H_2_O_2_ for 6 h and increased to 97.90 ± 5.0%, 93.81 ± 6.66%, 104.18 ± 5.01%, 100.18 ± 6.84%, and 96.09 ± 3.0% upon mangostanin treatment at 1.25, 5, 10, 20, and 40 μM, respectively. Mangostanol (10 μM) and demethylcalabaxanthone (5 μM) showed a preventive effect that was statistically significant only at one of the concentrations tested. On the other hand, the methanol extract of *G. mangostana* arils was similarly effective in the protection from oxidative cell damage. For this reason, we selected the methanol extract and mangostanin for the subsequent experiments at the most effective concentrations of 1 μg/mL and 10 μM, respectively.

### 2.4. Mangostanin Prevents the Generation of Intracellular Reactive Oxygen Species (ROS)

**Figure 5:**
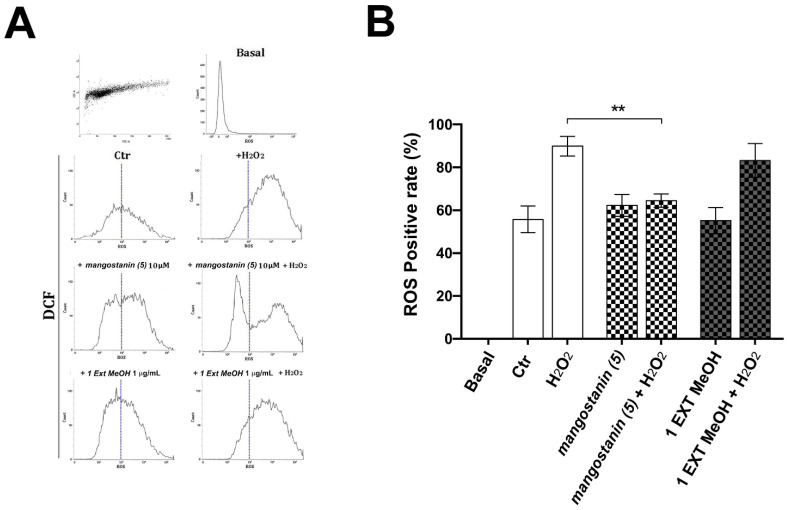
Mangostanin prevents the intracellular generation of ROS. HaCaT cells were treated with 200 μM of H_2_O_2_ with or without methanol extract of *G. mangostana* arils (1 μg/mL) or mangostanin (10 μM) for 6 h. Cells were incubated with the fluorescent dye for 30 min, and the mean fluorescence intensity was analyzed by flow cytometry. Representative cytofluorimetric histogram profiles are shown in (**A**), while the bar graph (**B**) reports the % of positive cells (ANOVA, ** *p* < 0.01 vs. control).

Keratinocytes were pretreated with mangosteen methanol extract or mangostanin for 18 h before adding 200 μM of H_2_O_2_ for 6 h to verify the protective effect on intracellular ROS generation. Production of ROS was evaluated by 2′,7′-dichlorofluorescein (DCF) measurement and monitored using flow cytometry. As demonstrated by an incremented fluorescence (Figure 5A), the intracellular ROS levels were induced by H_2_O_2_ treatment for 6 h. The percentage of cells with increased ROS production was 89.88 ± 4.6% in the H_2_O_2_-treated group, which was decreased only by mangostanin 10 μM pretreatment down to 64.4 ± 3.11% as shown in the graph (Figure 5A,B). Compared to the treatment with the methanol extract of *G. mangostana* arils (1 μg/mL), results showed that only mangostanin pretreatment significantly decreased the production of intracellular ROS following H_2_O_2_ exposure.

### 2.5. Mangostanin Prevents the Activation of Cell Death Molecular Pathways

**Figure 6:**
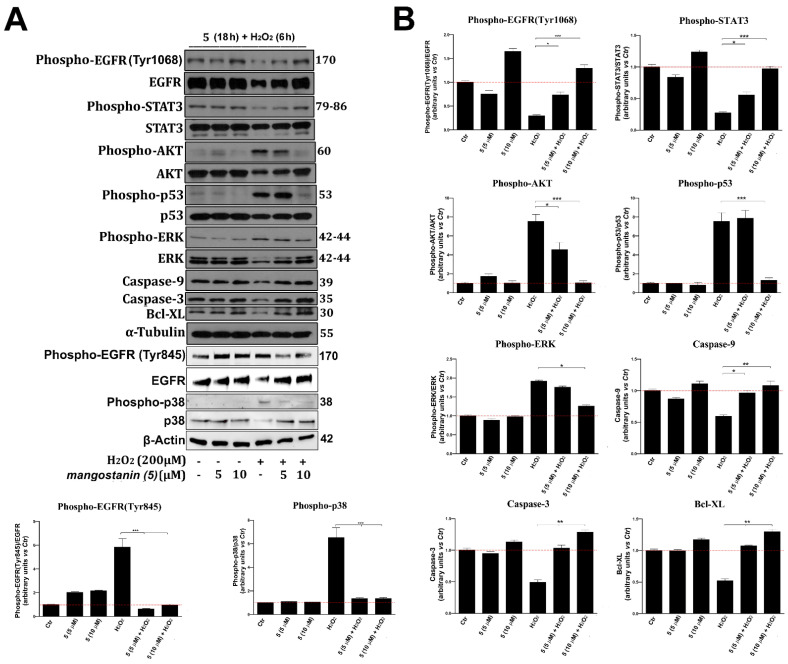
(**A**) WB analysis of EGFR, STAT3, AKT, p53, p38, ERK (total and phosphorylated), Caspase-9, Caspase-3, and Bcl-XL in whole cell lysates from keratinocytes cultured for 18 h with or without mangostanin (5 and 10 μM) and H_2_O_2_ (200 μM) for 6 h. As protein loading housekeeping, α-tubulin or β-actin were used. A representative WB of three different experiments performed with similar results is showed. (**B**) Histograms represent means ± SD in densitometry units of scanned immunoblots from the 3 different experiments (ANOVA, * *p* < 0.05, ** *p* < 0.01, *** *p* < 0.001, vs. control).

Mangostanin pretreatment significantly decreased the generation of intracellular ROS, and for this reason, we investigated the expression of proteins, such as epidermal growth factor receptor EGFR, protein kinase B (AKT), extracellular regulated protein kinase (ERK), p38 mitogen-activated protein kinases (p38 MAPK), p53, and signaling pathways which are implicated in the control of cell response to external damage induced by free radicals and in apoptosis activation [30,31,32,33]. Therefore, we determined by immunoblot the phosphorylation status of these proteins in cells pre-treated for 18 h with mangostanin (using both the more effective 10 μM concentration and the immediately lower one, 5 μM) and subsequently exposed to H_2_O_2_, as above described. We observed that the pre-treatment with mangostanin (10 μM) significantly prevented the phosphorylation and hence the activation of AKT, ERK, p53, p38 MAPK and EGFR (Tyr845), which are recognized markers of oxidative stress response. At the same time, the pre-treatment with mangostanin prevented the degradation of EGFR and the shutdown of its phosphorylation on Tyr1068, and hence of downstream signal transducer and activator of transcription-3 (STAT3) signaling, an index of cell survival and proliferation [34] (Figure 6A,B). Furthermore, mangostanin (10 μM) pre-treatment prevented the reduction of the total form of proteins, such as the caspases 3 and 9 and mitochondrial protein Bcl-XL involved in the induction of apoptosis [35], so preserving vitality.

## 3. Discussion

The biological relevance of the properties of the mangosteen fruit is widely known in natural medicine. It is reported as a natural regulator of biological functions, thanks to the antioxidants, anti-inflammatory and anticancer properties observed [8,9,10,11,12,13,14,15,17,18,19]. The unique nutritional composition of mangosteen could have a major impact in the design of novel food supplements [36,37]. At the moment, the greatest interest in this bioactive product is focused on its use as whole extract in many cosmeceutical products already available on the market. Despite its commercial success, its pharmacological efficacy in the field of cosmetic dermatology is yet not supported by in-depth scientific research. Furthermore, due to the high number of components, it is not yet clear which part of the phyto-complex or single molecule is the main responsible for the observed effects. Therefore, in our study, we evaluated the effect of different organic extracts of mangosteen and purified compounds on human keratinocytes, a well characterized cell line widely employed as an in vitro model of skin damage, for evaluating the dermatological properties of mangosteen.

Purified compounds were isolated from chloroform and methanol extracts of mangosteen arils and shells. Chloroform and methanol extracts of mangosteen arils showed a very similar HPLC profile, which led to the isolation of compounds **1**–**5**. The remaining compounds (**6**–**12**) were isolated in the chloroform and methanol extracts of mangosteen shells which afforded also compounds **2**, **3**, and **5**.

The lack of cytotoxicity from both the extracts and isolated compounds has been confirmed. Considering that numerous tests show how oxidative stress plays a key role in the pathogenesis of various diseases and skin aging, and that the regulation of ROS levels is fundamental for normal skin homeostasis [36,38], our experiments were aimed at demonstrating whether mangosteen was able to restore or prevent the oxidative damage induced by H_2_O_2_. We describe, for the first time, the biological effects of mangostanin in in vitro cultures of epidermal keratinocytes, comparing its activity with total extract and other xanthones. Indeed, we demonstrated that mangostanin was able to both rescue cells from H_2_O_2_-induced cytotoxicity and prevent cytotoxic damage caused by exposure to H_2_O_2_. Mangostanin, a xanthone purified from mangosteen extract, is therefore ideal for use in cosmeceutical formulations as it is more effective than whole mangosteen extract. The result obtained by the quantitative analysis of mangosteen arils highlighted a mangostanin (**5**) content of 106.4 mg per gram of chloroform extract.

As said before, mangosteen fruit extract has been shown to have elevated antioxidant effects as well as an anti-inflammatory potential, which can be beneficial to the vitality of various organs. For example, different compounds have been demonstrated to protect the hepatic tissue from the oxidative damage that may result from the drug detoxification process. Xanthones have been shown to prevent NfkB and MAPK activation in mice cell lines, attenuating cellular damage. In a similar manner, α-mangostin prevented the increase of pro-inflammatory cytokines, such as IL-6, interleukin-1β, and TNF-α after treatment with APAP and inhibited the increase of nitric oxide synthase (iNOS) expression, which further protects the liver tissue. Two other main compounds of mangosteen, isogarcinol and γ-mangostin, have been shown to exhibit a hepatoprotective effect by increasing anti-oxidative enzymes, such as SOD and glutathione, as well as inducing the expression of NRF2 (nuclear factor erythroid 2-related factor 2), a protein which regulates other anti-oxidative enzymes, such as heme oxygenase-1 and SOD2. Additionally, γ-mangostin can also upregulate SIRT1 (silent mating type information regulation 2 homologs 1), which in turn reduces ROS production from mitochondrial activity [39].

**Figure 7:**
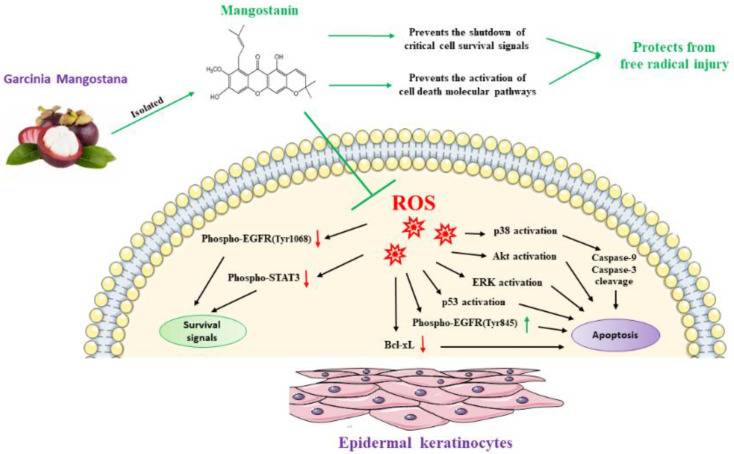
The xanthone mangostanin, isolated from *Garcinia mangostana* fruit, showed different protective effects against the cytotoxicity exerted from H_2_O_2_. Briefly, we showed that mangostanin prevents the shutdown of critical cell survival signals as the phosphorylation of EGFR on Tyr1068, STAT3, prevents the activation of p38, p53, AKT, ERK, the phosphorylation of EGFR on Tyr845, and the cleavage of caspase-9 and caspase-3, thus increasing cell vitality.

In our study, we showed that mangostanin has antioxidant potential and we supported these data at the molecular level by demonstrating that pretreatment with mangostanin not only decreased the generation of intracellular reactive oxygen species but prevented the activation of AKT, ERK, p53, cellular pathways underlying cell damage, and activation of apoptosis [29,30,31,32,33,34,35], as shown in Figure 7. Among the different markers shown here, Akt kinase, in particular, plays a central role in the cellular response against oxidative stress, specifically for H_2_O_2_-induced ROS. Increased Akt expression has been shown to be an antioxidant defense mechanism, and exogenous H_2_O_2_ has been reported to activate the PI3K/Akt pathway, which in turn controls apoptosis through the modulation of Bad/BcL-XL proteins [40]. Here, in fact, we showed how mangostanin can protect against oxidative stress by neutralizing the cell damage inflicted by ROS generated by H_2_O_2_ pre-treatment. The absence of ROS-induced damage allows cell survival and deactivation of the apoptosis pathway, a phenomenon that we also showed with the inhibition of ERK, p53 and the reduction of caspase-9 and caspase-3 cleavage.

We also investigated whether mangostanin could prevent p38 MAPK activation, induced by oxidative stress. As described in the literature, p38 acts as a nexus for signal transduction and has a central role in numerous biological processes [41]. It plays a vital role as signal transduction mediator for inflammation, cell cycle arrest, induction of cell death and senescence in several cell types. Therefore, it is recognized that the molecules that prevent the activation of p38 can have an important antioxidant role, also useful for minimizing the side effects of therapies in specific disease contexts [41,42]. In our experimental model, mangostanin at both tested concentrations, 5 μM and 10 μM, was able to significantly reduce (at 5 μM) and inhibit (at 10 μM) the activation of p38, thus preserving viability and proving to be a powerful antioxidant molecule.

The ability of mangostanin to inhibit p53 activation following oxidative stress was also evaluated in the present work. Indeed, p53 is a redox-active transcription factor that organizes and directs cellular responses in the face of a variety of stresses that lead to genomic instability, which acts by initiating DNA fragmentation to induce apoptosis, thus preventing aberrant cell proliferation [43].

ROS acts both as an upstream signal that triggers p53 activation and as a downstream factor that mediates apoptosis. In addition, p53 is also involved in an increasing number of diseases associated with aging [43,44]. We have shown that mangostanin, especially at a concentration of 10 μM, completely prevents the activation of p53 following oxidative stress. Therefore, a molecule such as mangostanin, by neutralizing the generation of ROS and preventing the activation of p53, can undoubtedly prevent aging and cell death.

The inhibition of p53 activation, thanks to mangostanin treatment, was also supported by a preservation of the total form of both Caspases 3 and 9, whose cleavage and activation is known to be a marker of apoptosis induced by oxidative stress or by various stressful conditions [29].

Finally, we evaluated EGFR expression and its phosphorylation on specific tyrosines. Indeed, it is known that not all EGFR phosphorylations mediate the same cellular response but, based on the phosphorylation positioning on the amino acid sequence, they can be both an indication of proliferation or viability or of response to oxidative stress. Recent studies show that EGFR phosphorylation on tyrosine 845 occurs following oxidative stress [45], while phosphorylation on tyrosine 1068 indicates cell proliferation and viability.

Therefore, in our work, we evaluated the expression of both phosphorylations (Tyr845 and Tyr1068) after treatment with H_2_O_2_ and in pretreatment with mangostanin. Treatment with mangostanin (5 and 10 μM) was able to inhibit EGFR phosphorylation on tyrosine 845, supporting previously obtained data, which once again demonstrates that mangostanin has antioxidant activity. At the same time, we have shown that mangostanin is able to prevent not only the degradation of EGFR, a protein necessary to preserve vitality and perform physiological functions, but also to preserve the phosphorylation of the receptor on tyrosine 1068, known to mediate signaling of cell proliferation and viability. Therefore, mangostanin proves to have a double activity following oxidative stress, capable of preventing the activation of cell stress and cell death pathways and at the same time preserving cellular vitality and proliferation.

Thanks to what has been carried out, our study provides evidence that mangostanin can protect the skin from the action of free radicals, thus preventing skin aging. These data, obtained in accordance with recent studies, show how mangosteen fruit extracts can be used as photoprotective agents against UVB damage [22,38], to improve skin elasticity and to control inflammatory skin diseases [23,46], configuring the mangosteen extract and, in our specific case, the mangostanin as a promising bio-active compound in the cosmetic sector. As evident in the HPLC-UV profiles, mangostanin represents not only one of the main compounds present in mangosteen aril extracts, but its presence in the shells highlights how the mangosteen by-products can also be a source of this biologically active compound.

Our study is in line with recent scientific works that aimed to evaluate not only the antioxidant activity of the total mangosteen extract, obtained with various extraction methods, but also to isolate its bioactive compounds.

Indeed, in recent years several studies have tried to implement the extraction methods to obtain mangosteen extracts with an increasingly greater antioxidant activity. The drying and extraction processes have been improved [47], fresh fruit has been used to try to avoid drying [8], different solvents and microwave-assisted extraction were used [48,49].

To date, the antioxidant activities of mangosteen extracts have been mainly associated to the prenylated xanthones α-, β-, and γ-mangosteens, which are also the main compounds represented in the fruit [50,51,52].

It has been shown that both mangosteen extract and α-mangosteen can decrease the activity of myeloperoxidase and at the same time increase the activity of catalase and superoxide dismutase inhibiting the production of reactive oxide species and malonaldehyde [53], configuring them as useful in all diseases related to aging and oxidative stress [54].

In addition to antioxidant and scavenging activities, mangosteen extract also has anti-elastase activity. A-mangosteen showed potent anti-collagenase activity, while γ-mangosteen has potent anti-hyaluronidase and anti-tyrosinase activities providing evidence of the possible use of mangosteen extract and its compounds as antioxidants and anti-aging treatments [55].

Mangosteen extract, also based on the extraction conditions, shows not only similar but also additional activities compared to the isolated molecules most studied for the proven antioxidant activities. For this reason, newly identified xanthones, even if less represented in the fruit, such as mangostanin, could show a higher antioxidant activity than other well-known molecules, explaining at least in part the superior effect of the total extract.

This study employing HaCaT immortalized keratinocytes that have known limits to fully resemble skin needs to be validated in the future in primary keratinocytes, in order to account for donor-to-donor variability and in more sophisticated three-dimensional skin models [56]. To evaluate the topical availability of mangostanin in cosmeceutical formulations, it will be necessary to understand its penetration through the skin barrier and the resistance of the stratum corneum. Therefore, it is undoubtedly necessary to investigate the stability, skin permeation, topical bioavailability, and efficacy of the molecule. Certainly, the potential of mangostanin as a topical ingredient will be confirmed if new studies are conducted as skin permeation studies using both in vitro skin models and bioactive compound delivery system techniques (going up to the use of nanotechnology) as much as the evaluation of stability of formulations. Finally, further in vivo and clinical studies will be required to develop and validate novel nutraceuticals, pharmacological formulations, and cosmeceuticals.

## 4. Materials and Methods

### 4.1. Chemicals and Materials

NMR spectroscopic data were acquired in MeOH-d4 (99.95%, Sigma-Aldrich, St. Louis, MO, USA) on a Bruker DRX-600 spectrometer (Bruker BioSpin GmBH, Rheinstetten, Germany) equipped with a Bruker 5 mm TCI CryoProbe at 300 K. Data processing was carried out with Topspin 3.2 software.

We solubilized the extracts and pure compounds in DMSO (dimethyl sulfoxide) (≤0.01% when added to cell cultures). H_2_O_2_ and 2′,7′-Dichlorofluorescein were purchased from Sigma-Aldrich, Inc. (St. Louis, MO, USA). For western blot analysis, we used: mouse monoclonal antihuman α-tubulin, rabbit monoclonal antihuman phospho-EGF receptor (p-EGFR; Tyr1068), rabbit monoclonal antihuman phospho-EGF receptor (p-EGFR; Tyr845), rabbit polyclonal antihuman phospho-STAT3 (p-STAT3; Tyr705), rabbit monoclonal anti-human Phospho-Akt (p-Akt; Ser473), rabbit polyclonal antihuman phospho-p38 MAPK, rabbit polyclonal antibody to phosphorylated p53, rabbit monoclonal anti-human phospho-p44/42 MAPK (p-Erk1/2; Thr202/Tyr204), rabbit monoclonal anti-human EGF receptor, rabbit monoclonal anti-human STAT3, rabbit monoclonal anti-human Akt, rabbit monoclonal anti-human p44/42 MAPK (Erk1/2), rabbit monoclonal anti-human p38 MAPK, rabbit polyclonal anti-human p53, rabbit polyclonal anti-human caspase-3, rabbit monoclonal anti-human caspase-9, rabbit monoclonal anti-human Bcl-xL, and secondary HRP-linked goat anti-mouse or goat anti-rabbit IgG were purchased from Cell Signaling Technology (Danvers, MA, USA). Rabbit polyclonal anti-human β-actin was purchased from Abcam (Cambridge, UK).

### 4.2. Plant Material

The fruits of *Garcinia mangostana* were purchased from an online market in March 2017 and classified by Prof. V. De Feo (Department of Pharmacy, University of Salerno, Italy).

### 4.3. Extraction and Isolation Procedures of G. mangostana Arils and Shells

*Garcinia mangostana* arils (200g) were stored in the freezer at a temperature of −5 °C. After some days, the fruits were submitted to lyophilization to obtain 42.32 g, and the dried arils deprived of seeds were extracted at room temperature using solvents of raising polarity, such as petroleum ether (300 mL for 3 days, three times), CHCl_3_ (300 mL for 3 days, three times), and MeOH (300 mL × 3 days × 3 times). We obtained 0.37 g and 16.20 g of crude extracts of CHCl_3_ and MeOH, respectively, by filtration and evaporation of the solvent to dryness in vacuo.

*Garcinia mangostana* shells (370 g) were dried and extracted at room temperature with petroleum ether (1500 mL for 3 days, three times), CHCl_3_ (1500 mL × 3 days × 3 times), and MeOH at room temperature (1500 mL × 3 days × 3 times). The filtrates were concentrated under reduced pressure until elimination of CHCl_3_ and MeOH to obtain 13.62 g and 55.89 g of crude extracts, respectively.

The CHCl_3_ and MeOH extracts of *G. mangostana* arils (1 Ext CHCL3 and 1 Ext MeOH) and shells (2 Ext CHCL3 and 2 Ext MeOH) were analyzed by an RP-HPLC-UV system on an Agilent 1260 Infinity system (Agilent Technologies, Palo Alto, CA, USA), equipped with a binary pump (G-1312C), and a UV detector (G-1314B) with a Phenomenex C_18_ Synergi-Hydro-RP (250 mm × 10 mm, 10 μm) column; the elution gradient was executed using water with 0.1% formic acid as eluent A and acetonitrile with 0.1% formic acid as B at a flow rate of 2.0 mL/min. In particular, the HPLC gradient started at 5% B, and after 5 min % B was at 50%, after 15 min at 80%, and after 10 min at 87%. % B remained at 87% for 20 min, and finally after 10 min, it reached 100% and so remained for 20 min. The selected wavelength was 254 nm.

The chloroform extract (twenty injection of 2.5 mg each) of mangosteen arils yielded 1,7-dihydroxy-3-methoxy-2-(3-methylbut-2-enyl)-xanthen-9-one (**1**) (2.3 mg, t_R_ = 30.9 min), 1,3,7-trihydroxy-2,8-bis-(3-methyl-2-buten-1-yl)-9H-xanthen-9-one (**2**) (1.5 mg, t_R_ = 33.3 min), *α*-mangostin (**3**) (2.9 mg, t_R_ = 34.8 min), demethylcalabaxanthone (**4**) (1.1 mg, t_R_ = 45.0 min), and mangostanin (**5**) (3.2 mg, t_R_ = 46.1 min). The methanol extract submitted at HPLC purification in the same conditions afforded compounds **1**–**5**.

The chloroform extract (twenty injection of 2.5 mg each) of mangosteen shells yielded mangostanol (**6**) (1.8 mg, t_R_ = 16.2 min), garcinone D (**7**) (2.3 mg, t_R_ = 24.0 min), *γ*-mangostin (**8**) (2.0 mg, t_R_ = 29.5 min), gudraxanthone (**9**) (1.2 mg, t_R_ = 31.0 min), 8-deoxygartanin (**10**) (2.2 mg, t_R_ = 32.0 min), 1,3,7-trihydroxy-2,8-bis-(3-methyl-2-buten-1-yl)-9H-xanthen-9-one (**2**) (1.3 mg, t_R_ = 33.3 min), *α*-mangostin (**3**) (5.2 mg, t_R_ = 34.8 min), garcinone E (**11**) (2.1 mg, t_R_ = 36.0 min), mangostanin (**5**) (2.1 mg, t_R_ = 48.4 min), and *β*-mangostin (**12**) (0.2 mg, t_R_ = 49.7 min). The methanol extract in the same conditions afforded compounds **3**, **6**–**8** and **10**–**12**.

The purity of these compounds (>99%) was determined by HPLC analysis.

### 4.4. Quantitative Analysis of Mangostanin (**5**)

The quantitative determination of mangostanin (**5**) in the chloroform extract of mangosteen arils was performed by HPLC-UV. HPLC conditions were the same as used for qualitative analysis with slight modifications. Mangostanin (**5**) was quantified using the calibration curve of the corresponding standard. Standard stock solutions were prepared by stepwise dilution with 5 mg of mangostanin (**5**) in 5 mL of methanol, to reach the concentration range of 10 to 100 μg/mL. The calibration curve was obtained by plotting the UV peak area of external standard against the known concentration of the compound; each concentration of standard solution was analysed in triplicate. The amount of mangostanin (**5**) was expressed as milligrams per gram of extract as the mean of triplicate determinations.

### 4.5. Cells

We grew the human immortalized keratinocytes (HaCaT) in DMEM (Dulbecco’s modified Eagle’s medium) purchased from GIBCO (Grand Island, NY, USA) and supplemented as described in detail elsewhere, using 2 mM L-glutamine, 50 ng/mL, streptomycin, 50 units/mL of penicillin, and 10% heat-inactivated fetal bovine serum [28]. Cell cultures were maintained at 37 °C in a humidified 5% CO_2_ atmosphere.

### 4.6. Determination of Cell Viability—MTT Assay

HaCaT cells (6 × 10^3^/well) were grown for 24 h into 96 multi-well plates before the addition of each molecule at the indicated concentration and then further cultured for an additional 24, 48, or 72 h at 37 °C. Then, we employed the MTT (3-(4, 5-dimethylthiazolyl-2)-2, 5-diphenyltetrazolium bromide) tetrazolium salts assay to determine cell viability, as described in detail elsewhere [57]. Briefly, 10 μl of MTT solution was added (5 mg/mL, in water) to each well. After an incubation time of 4 h, the formazan crystals were dissolved in 100 μl of solubilization solution (10% Triton X-100 and 0.1 N HCL in isopropanol). A microplate reader (ThermoScientific, Basingstoke, UK) was used to detect the absorbance intensity at 595 nm. We have performed all experiments in triplicate and expressed the relative cell viability as % with respect to the untreated control cells.

### 4.7. Determination of Reactive Oxygen Species (ROS)—Flow Cytometric Assay

Production of reactive oxygen species (ROS) was evaluated by 2′7′-dichlorodihydrofluorescein diacetate dye staining, converted by cellular esterase to H_2_DCF and subsequently oxidized to highly fluorescent 2′,7′-dichlorofluorescein (DCF). At the end of the treatment with H_2_O_2_, methanol extract of *Garcinia mangostana* arils (1 μg/mL) and mangostanin (10 μM), cells were washed with ice-cold PBS 2% FBS and loaded with 20 μM of H_2_DCFDA for 30 min. The cells were washed again with PBS 2% FBS, acquired by flow cytometry where at least 10,000 events were collected and next analyzed by Cell-Quest Pro software (BD Biosciences, San Jose, CA, USA) [58].

### 4.8. Western Blot (WB) Assay

Cells were grown in p60 tissue culture plates at a density of 2 × 10^4^ cells/cm^2^ for 24 h. Cells were then treated with mangostanin, H_2_O_2_, or their combination, as indicated. Then, the cells were washed with PBS, collected, and lysed in cold RIPA (radioimmunoprecipitation assay) lysis buffer (50 mM Tris-HCl, 10 mg/mL aprotinin, 2 mM phenylmethylsulfonyl fluoride, 0.5% Triton X-100, 0.5% deoxycholic acid, 150 mM NaCl, and 10 mg/mL leupeptin) and then assayed for (WB) by the procedure described in detail elsewhere [59].

### 4.9. Statistical Analysis

We performed the statistical analysis by GraphPad Prism 6.0 software for Windows (GraphPad software, San Diego, CA, USA). Data obtained from multiple experiments expressed as mean ± (SD) of four independent experiments conducted in triplicate were analyzed for statistical significance through 2-tailed Student t-test for independent groups or 1-way ANOVA followed by Tukey post-hoc correction for multiple comparisons. *p* values < 0.05 were considered significant. * *p* < 0.05, ** *p* < 0.01, and *** *p* < 0.001).

## 5. Conclusions

We provided for the first time the scientific evidence that mangostanin, a xanthone identified in the fruit of *Garcinia mangostana*, remarkably protects epidermal keratinocytes from the action of free radicals, demonstrating a route for possible pharmaceutical applications. Taken together, our findings showed the protective effects of mangostanin on epidermal keratinocytes. Pre-treatment with the substance significantly reduced H_2_O_2_-induced cytotoxicity and ROS production, prevented activation of p53, p38 MAPK, ERK and AKT, cleavage of Caspase-9 and Caspase-3, and shutdown of critical signals of cell survival, such as EGFR and STAT-3, thereby increasing cell viability. Furthermore, the ability to prevent skin aging brings the molecule to the attention of the cosmeceutical sector. Undoubtedly, further in vivo and clinical studies are needed to develop and validate novel cosmeceutical, pharmacological or nutraceutical formulations, based on this interesting molecule.

## Figures and Tables

**Table 1:** ^13^C NMR Data (150 MHz MeOH-*d*4) for Compounds **1**–**12**.

	1	2	3	4	5	6	7	8	9	10	11	12
1	159.9	161.0	161.8	153.4	150.4	156.4	161.0	161.5	157.0	157.0	161.8	161.4
2	112.3	110.8	111.4	104.7	105.3	105.0	111.3	110.8	112.3	112.3	110.6	111.0
3	165.8	163.7	163.8	160.9	160.3	162.4	163.8	163.0	165.5	165.5	163.8	163.8
4	90.4	92.5	92.9	94.4	94.5	94.4	92.8	92.4	90.8	90.8	92.8	92.9
5	119.4	116.4	102.5	123.4	102.7	102.1	102.3	100.6	147.8	147.8	114.3	102.5
5a	157.7	156.0	156.2	157.7	157.7	158.6	156.1	155.9	157.7	157.7	155.7	156.1
6	124.8	123.4	156.6	116.5	157.0	155.9	156.4	154.2	121.2	121.2	148.6	156.4
7	155.3	151.9	144.8	152.5	144.2	144.4	144.8	141.8	124.6	124.6	141.4	144.8
8	108.9	129.6	138.6	119.0	138.6	137.7	139.0	129.4	115.3	115.3	128.6	138.8
8a	121.8	119.6	112.0	129.0	111.3	115.1	111.5	111.8	122.3	122.3	114.0	112.0
9a	104.5	103.4	103.8	104.5	104.5	107.0	104.0	103.5	104.3	104.3	102.8	103.8
9	183.8	184.4	183.6	184.4	183.6	179.0	180.9	182.7	181.9	182.5	183.0	183.5
10a	150.0	151.8	158.3	152.7	157.7	156.8	157.2	154.0	146.5	146.5	152.2	157.9
1′	21.7	22.1	22.1	115.8	116.0	27.3	21.9	21.6	21.9	21.9	22.0	22.1
2′	123.5	123.4	123.8	128.1	128.3	69.3	123.6	123.6	123.0	123.0	124.0	123.9
3′	132.1	131.1	131.8	78.8	78.6	79.4	131.6	131.3	131.8	131.8	130.9	131.7
4′	17.5	17.2	17.6	28.1	27.9	19.9	17.7	17.4	17.5	17.5	17.4	17.8
5′	25.8	26.1	25.9	28.1	27.9	25.3	25.8	25.8	26.0	26.0	25.8	25.9
1″		26.5	26.9	26.5	26.3	26.9	23.0	26.0			26.7	26.5
2″		123.8	124.8	124.4	124.6	125.4	45.7	124.5			124.0	124.4
3″		131.0	132.0	131.4	131.5	131.8	71.6	131.2			131.2	132.0
4″		18.5	18.1	18.3	18.5	18.6	29.1	17.9			18.1	18.1
5″		26.1	25.9	26.2	25.9	26.3	29.1	25.5			25.8	25.9
1‴											23.3	
2‴											124.0	
3‴											131.8	
4‴											18.3	
5‴											28.0	
OCH_3_	56.1								56.0			56.0
OCH_3_			61.1		60.9	61.0	61.3					61.1

## Data Availability

Data is contained within the article and supplementary material.

## Supplementary Materials

The following are available online at https://www.mdpi.com/article/10.3390/ph15010084/s1, Figure S1: Evaluation of 1 Ext MeOH, 1 Ext CHCl3, 2 Ext MeOH, 2 Ext MeOH, 1, 2, 3, 4, 5, 6, 7, 8, 9, 11, and 12 effects in HaCaT Cells.
