# Supplementary material for: Mangostanin, a Xanthone Derived from Garcinia mangostana Fruit, Exerts Protective and Reparative Effects on Oxidative Damage in Human Keratinocytes
Source: Pharmaceuticals (Basel). 2022 Jan 11;15(1):84. doi: 10.3390/ph15010084 (PMC8780152; doi:10.3390/ph15010084)
Supplement: Supplementary file 1 [file pharmaceuticals-15-00084-s001.zip › pharmaceuticals-1450636-supplementary.pdf]

Supplementary information

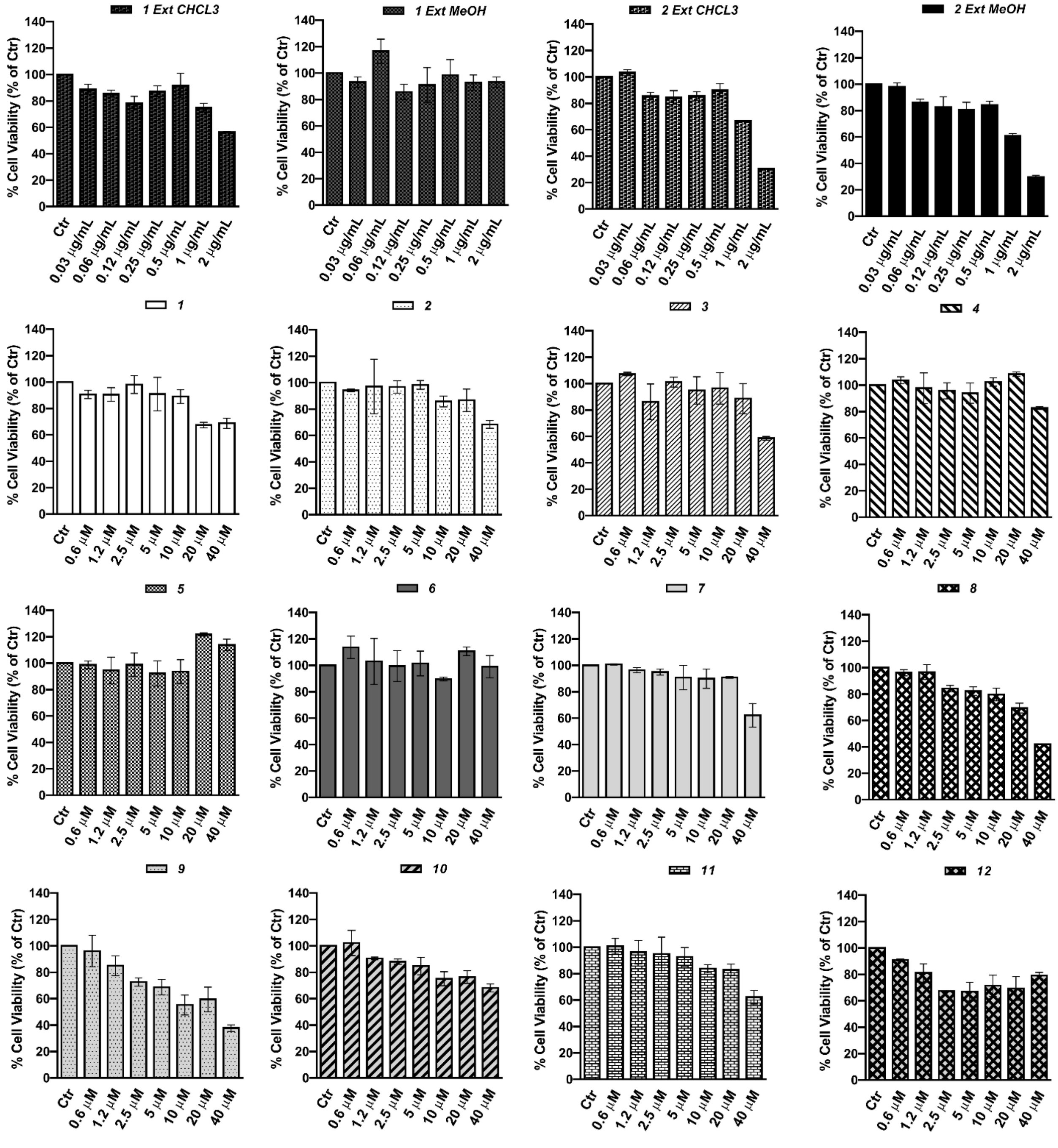

Figure S1. Evaluation of 1 Ext MeOH, 1 Ext CHCl3, 2 Ext MeOH, 2 Ext MeOH, 1, 2, 3, 4, 5, 6, 7, 8, 9, 11, and 12 effects in HaCaT Cells. HaCaT cells were cultured for 48 h in the presence of the indicated concentrations (0-40 µM) of individual substances or extracts (µg/mL) before MTT assay. Results are expressed as means ± SD of independent experiments performed in triplicate and reported as percentage *vs*. the untreated control).
